# Infrared thermographic emissivity of heat generated during enamel versus dentin preparation: a linear regression model

**DOI:** 10.1038/s41598-026-55686-4

**Published:** 2026-06-05

**Authors:** Franco Mauricio, Cesar Mauricio-Vilchez, Mabel Huaman-De la Cruz, Diego Galarza-Valencia, Luzmila Vilchez, Fran Espinoza-Carhuancho, Frank Mayta-Tovalino

**Affiliations:** 1 Research, Innovation and Entrepreneurship Unit, Faculty of Dentistry, Universidad Nacional Federico Villarreal, Lima, Peru; 2 EVIDENTIA Research Group, Universidad Naciional Mayor de San Marcos, Lima, Peru; 3 Vicerrectorado de Investigación, Universidad San Ignacio de Loyola, Lima, Peru

**Keywords:** Enamel, Temperature mapping, Dentin, Thermography, Health care, Materials science, Medical research

## Abstract

The preparation of dental cavities generates heat through friction that can affect the internal structure of the tooth, especially the dentin. This study explores the infrared thermal emissivity in enamel and dentin, with and without irrigation, using different types of rotary burs to identify safer practices during dental procedures. An in vitro experimental study was developed following the CRIS Guidelines in the Orthodontics Laboratory at UNFV. Human premolars extracted for orthodontic reasons (*n* = 20 per group) were used and randomly assigned to conditions with or without irrigation in the enamel and dentin. The cavities were prepared with round and cylindrical burs using a KaVo high-speed turbine. The surface temperatures were recorded with a Fluke TiS55 + thermal imaging camera, calibrated and focused at 1 m. Statistical analysis included Student’s t-tests and multiple linear regression models in Stata 17.0. Dentin without the use of irrigation had a higher thermal emissivity than enamel (up to 39.5 °C compared to enamel 32.05 °C), indicating that dentin has greater sensitivity to friction. Irrigation was able to reduce the temperature to approximately 22 °C in both tissues with no significant difference between bur types. The statistical model demonstrated that for every 1 °C increase in enamel temperature, dentin temperature rose by an additional 0.40 °C (*p* < 0.05). In contrast, variations in bur geometry did not exert a significant influence on dentin heat generation, underscoring that tissue response was primarily driven by thermal transfer rather than instrument design. Irrigation demonstrated strong efficacy as a thermal regulator, significantly reducing dentin temperature during cavity preparation. These results underscore the critical role of irrigation in preserving the integrity of internal dental tissues—particularly in clinical scenarios where direct cooling is not applied.

## Introduction

The dental pulp is a richly vascularized tissue vulnerable to damage during cavity preparation and restorative procedures. Thermal injury poses a particular risk due to temperature increases transmitted through enamel and dentin, potentially compromising pulp health^1^. Although dentin exhibits low thermal conductivity, the likelihood of pulp damage rises with deeper preparations where dentinal tubule exposure is greater^2^. Preserving pulp vitality after intervention hinges on the survival and reparative capacity of odontoblasts. Minimizing irritation during restorative procedures is therefore critical to support the tissue’s natural healing mechanisms^3^.

Several clinical procedures during dental treatment can result in the generation of heat and increased intrapulpal temperature. Tooth cutting with high-speed handpieces, the exothermic reactions taking place during the polymerization of restoratives, both light-cured and self-cured, and finally the process of polishing are such steps. Although these assume considerable clinical importance, little evidence is available regarding the factors which do exert an influence on the rise in pulp temperature^4^. To minimize these issues, dental handpieces should feature a comfortable design that balances size and weight, a head approximately matching the size of the teeth, adequate power and rotational speed, good visibility with sufficient lighting, and—most importantly—an efficient cooling system^5,6^. These cooling features are essential as dental cutting generates friction and heat between the bur and the tooth surface^7^. High-speed handpieces, while essential in restorative dentistry, can add thermal risk, as the concurrences of speed, torque, and bur type can increase the amount of friction at the tooth surface. The burs should be cooled properly and effectively; a fast-moving bur too hot to touch can produce extreme temperatures when effective cooling is lacking, such temperatures exceed the tolerance of enamel and dentin and heat is conveyed to the pulp chamber. Mechanical and/or visibility restraints may cause increased pressure by the operator^3–6^.

Excessive temperature rises may also induce pulp inflammation and irreversible damage, representing another aspect of dentin and enamel alteration^6^. According to Zach and Cohen, a temperature increase of 5.5 °C is commonly used as the limit in vitro temperature research. Any variation exceeding this threshold is potentially harmful to pulp health^8^. Damage to the dental pulp can be caused by factors such as heat, especially if water cooling is inadequate, air and pressure applied to the dentin, particularly when removing it close to the pulp^9^. In addition, the heat generated during dental procedures is influenced by factors such as the size and type of bur, torque, instrument abrasiveness, pressure exerted, and amount of tissue removed^10^. Therefore, it is essential that the dentist control these elements to minimize the temperature increase^7^.

In addition, the number and orientation of the handpiece’s cooling outlets must be considered, as they should direct the flow toward the tip of the bur^11^. The use of air and water spray is essential during high-speed dental preparation, regardless of the pressure applied or the type of bur, such as diamond, steel, or carbide^7^. This technique also prevents excessive drying and contributes to a better cut^12^. On the other hand, when cooling is not used, the bur can reach between 200,000 and 400,000 rpm, generating temperatures of up to 240 °C at the enamel-dentin junction^13^.

Therefore, the purpose of this research was to evaluate the infrared thermographic emissivity of the heat generated during enamel and dentin preparation using a linear regression model.

## Methods

### Study design

**Fig. 1:**
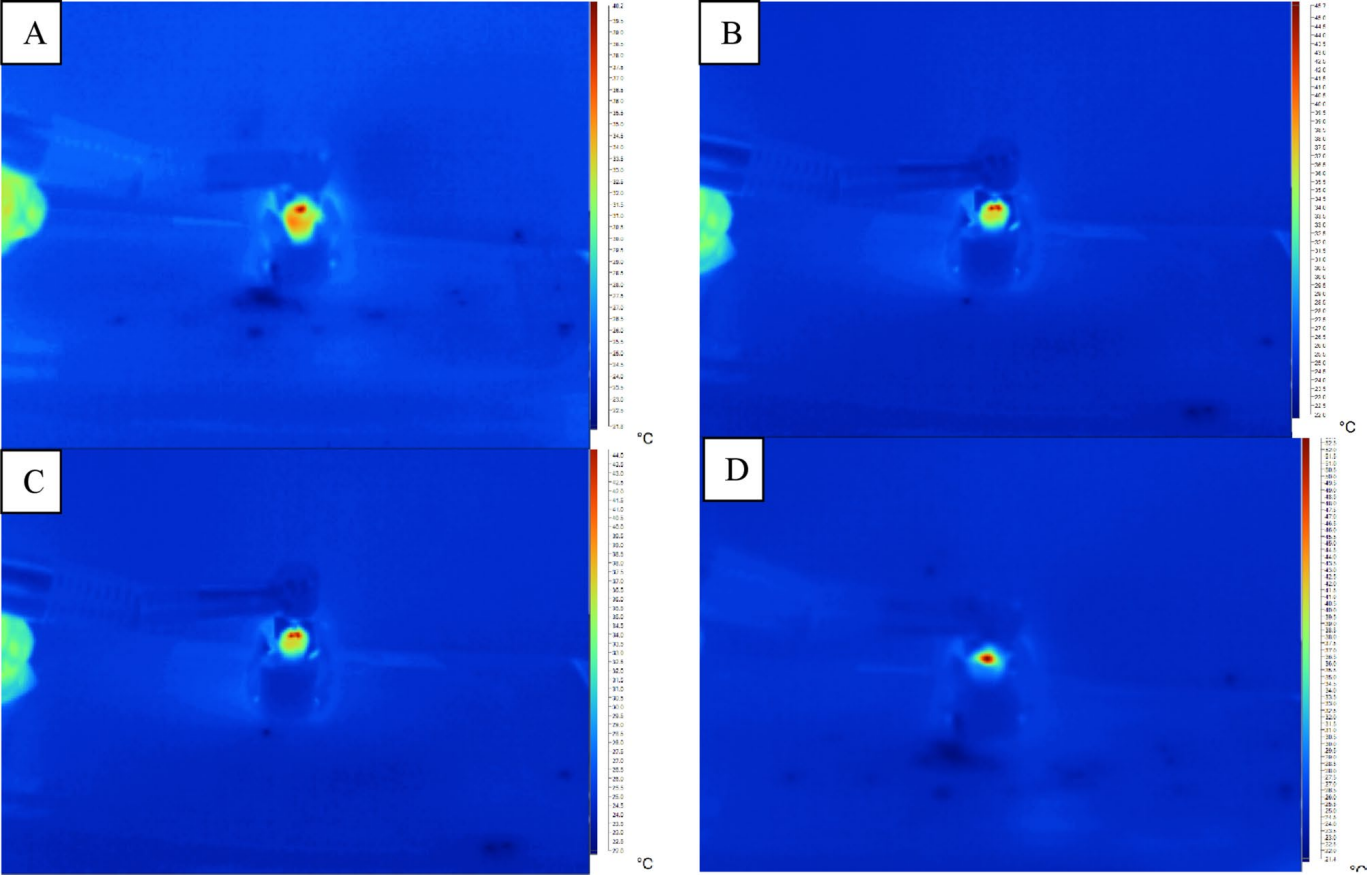
Thermography in Enamel and Dentin according to the type of bur without irrigation. (**A**) Heat generation in enamel with the round drill; (**B**) Heat generation in enamel with the cylindrical drill; (**C**) Heat generation in dentin with the round drill; (**D**) Heat generation in dentin with the cylindrical drill. All without irrigation.

An in vitro experimental study was carried out to compare the infrared thermographic emissivity of heat generated during cavity preparation in enamel and dentin. The findings were reported in accordance with the CRIS Guidelines (Checklist for Reporting In-vitro Studies)^14^, ensuring methodological transparency and consistency. The study took place at the Orthodontics Laboratory of the Faculty of Dentistry, Federico Villarreal National University, in Lima, Peru (Fig. 1).

### Sample size calculation

**Fig. 2:**
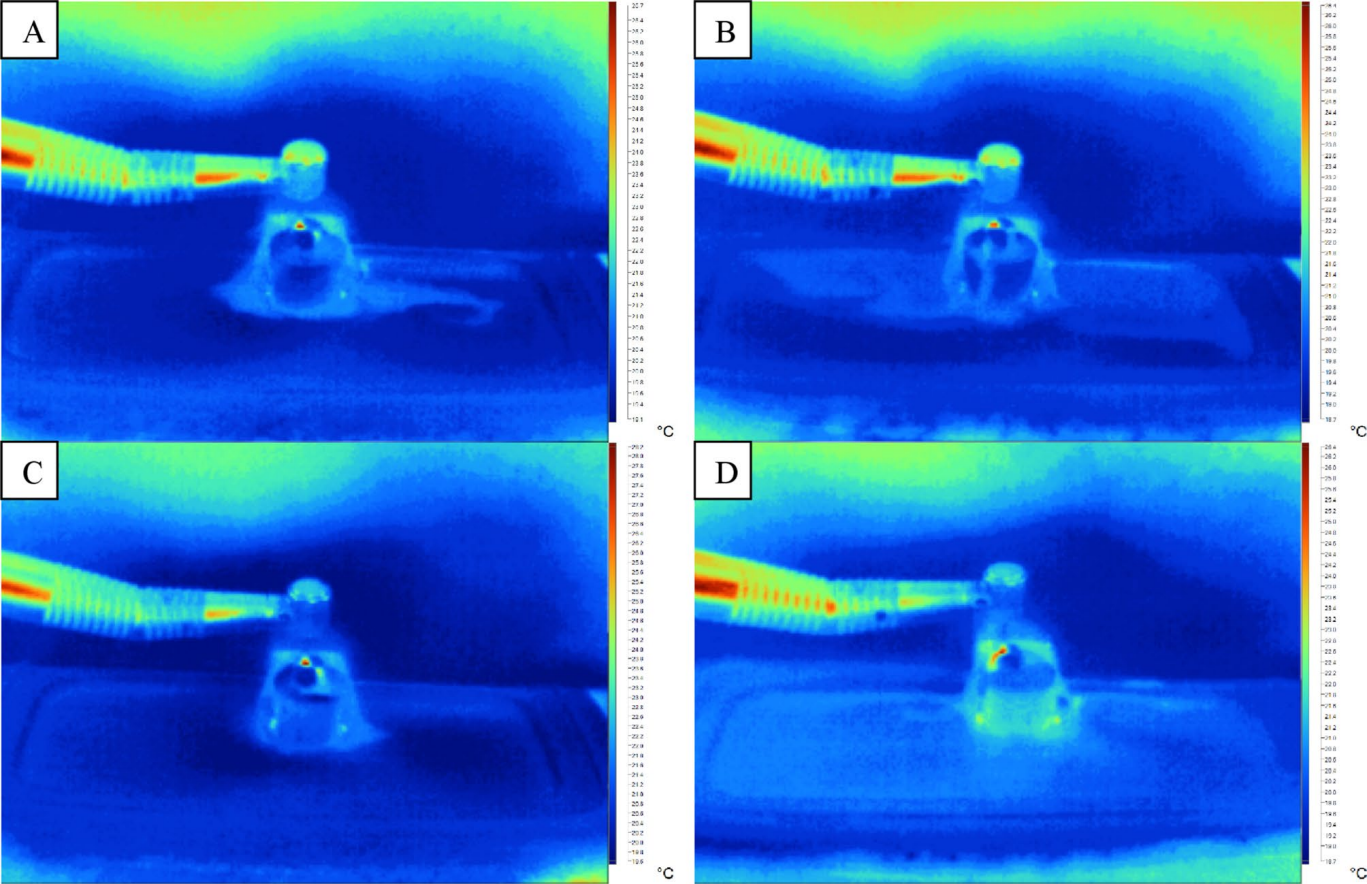
Thermography in Enamel and Dentin according to the type of bur with irrigation. (**A**) Heat generation in enamel with the round drill; (**B**) Heat generation in enamel with the cylindrical drill; (**C**) Heat generation in dentin with the round drill; (**D**) Heat generation in dentin with the cylindrical drill. All with irrigation.

The sample size was determined based on a statistical power of 80% and a significance level of 0.05 for the determination of differences ≥ 2 °C between groups, which yielded a total of extracted human teeth, perfectly distributed in the experimental groups (*n* = 20 per group in the sample power analysis). All calculations were performed using the mean comparison formula with the Stata 17.0 program (Fig. 2).

### Allocation

The dental specimens were randomly distributed into four experimental groups according to tissue type and the presence or absence of additional water cooling: enamel without irrigation, enamel with irrigation, dentin without irrigation, and dentin with irrigation. Each group was then further divided according to the type of rotary instrument used—round bur or cylindrical bur—to achieve a balanced allocation that would ensure control of the cutting geometry.

### Obtaining and preparing the teeth

Upper and lower human first and second premolars extracted for clinical reasons unrelated to the objectives of the study, i.e., for orthodontic indications, were collected. The objects were donated by the Orthodontics Department of the UNFV School of Dentistry, preserved in a saline solution, and disinfected in accordance with the ethical protocol approved by the Institutional Committee. Subsequently, the organic remains present in each of the teeth were removed, and acrylic blocks were created for each tooth to obtain controlled and standardized access for cavity preparation.

### Preparation for dental cavities

Cavity preparations were made using a high-speed turbine (KaVo 636 CP, Compact Torque, Germany) with Odontho diamond burs (Microdont, Brazil) Two series of 1.6 mm FG burs were used (Microdont) - round bur (FD-R1016; diamond medium grain; maximum speed 450, 000 rpm), for cutting enamel and dentin and a cylindrical bur (FD-C1016; diamond medium grain; maximum speed 450,000 rpm). All burs were sterilized in an autoclave just prior to use, and water cooling was standardized using saline solution to ensure fluid viscosity and thereby consistent temperature control. The cavities were prepared according to the design of Black, Class I. As nearly as possible, a standard procedure was carried out: each preparation limiting time to 1–2 min, at an equal and uniform pressure, with bur perpendicular to the occlusal surface and moved slowly forward and backward to avoid excessive localized friction. Tissue was removed with light short, interrupted cutting instead of continuous prolonged contact so as to keep the tooth constantly cooled. Less variability might be introduced by the operator in this type of control environment and consistency in temperature of the cavity preparation were guaranteed.

### Thermographic recording

The procedure for determining the thermographic emissivity was carried out under standardized conditions to ensure the measurement conditions of the surface temperatures in the enamel and dentin. A Fluke thermographic camera (TiS55+) (infrared resolution of 256 × 192 px; 3.1 mp) was used, which was previously calibrated and with the manual focus system locked at 1 m, which kept the distance between the camera and the study site constant in each shot. For enamel, an approximate emissivity value of 0.96 was used, and for dentin, a value close to 0.92 was used. These values were chosen based on specific literature and previous studies on the thermal properties of dental tissues, so that the surface heat generated in the cavity preparation could be represented with faithful approximation.

### Statistical analysis

The arithmetic means and standard deviations of the recorded temperatures were determined for each of the experimental groups, allowing the overall distribution of the results to be characterized. The Shapiro-Wilk test for normality was then applied to verify the assumption of normality before the parametric analyses. To compare temperatures between the groups, the variables were tissue type, irrigation use, and bur type. Independent-sample Student’s t-tests were used to test for differences between groups at a significant level. Ultimately, it was decided to construct a multiple linear regression model to identify the main predictors of the final dentin temperature, using the irrigation type, bur geometry, and previously recorded enamel temperature as covariates. All analyses were performed using Stata version 17.0. A p-value of *p* < 0.05 was considered indicative of significance.

## Results

The findings demonstrate a thermal difference between the enamel and dentin during tooth preparation when no additional water cooling was used. Dentin had high infrared thermal emissivity values (39.5 ± 2.17 °C with round bur; 37.9 ± 1.53 °C with cylindrical bur) than enamel (32.05 ± 1.98 °C; 29.4 ± 1.64 °C, respectively; the dentin appears to be mechanically more susceptible to friction. Water cooling significantly reduced the temperature of enamel and dentin in the range of 21.37 °C and 23.92 °C. There were no overall differences with bur type or tissue, and no statistically significant differences. The Shapiro-Wilk normality test was employed for the distributions of all variables with all distributions considered normal (*p* > 0.05), thus valid parametric statistical models may be applied. (Table 1)

**Table 1:** Infrared Thermographic Emissivity of Heat Generated During Enamel versus Dentin Preparation.

	Without irrigation				p*	Witht irrigation				p*
	Drill					Drill				
	Round		Cylindrical			Round		Cylindrical		
	Mean	SD	Mean	SD		Mean	SD	Mean	SD	
Enamel	32.05	1.98	29.4	1.64	> 0.05	21.49	1.08	23.52	1.64	> 0.05
Dentin	39.5	2.17	37.9	1.53	> 0.05	21.37	0.62	23.92	0.96	> 0.05

p*: Normality test (Shapiro–Wilk test).

Dentin without irrigation showed significantly higher temperature values at 38.7 ± 0.32 °C than enamel temperature at 30.7 ± 2.23 °C. These comparison results showed significant differences *p* = 0.001, indicating that it is a more thermally sensitive internal tissue than enamel to mechanical friction. Irrigated dentin and irrigated enamel showed comparable temperature means with drastically lower values of 22.5 °C (enamel) and 22.64 °C (dentin), which was likely a protective effect from the removal of friction heat. The results showed no significant differences in bur type (round vs. cylindrical), but each bur both presented satisfactory thermal values and non-significant p-values (*p* > 0.05) indicating no differences in estimated temperature values between bur type. (Table 2)

**Table 2:** Comparison of Heat Generated During Enamel versus Dentin Preparation.

		Temperature		Confidence intervals	P**
		Mean	SD		
Enamel	Without irrigation	30.7	2.23	30.01 to 31.44	0.001
	With irrigation	22.5	1.71	21.95 to 23.05	
Enamel	Round drill	26.77	0.88	24.98 to 28.55	0.770
	Cylindrical drill	26.46	0.53	25.38 to 27.55	
Dentin	Without irrigation	38.7	0.32	38.05 to 39.35	0.001
	With irrigation	22.64	0.24	22.15 to 23.13	
Dentin	Round drill	30.43	1.47	27.45 to 33.41	0.800
	Cylindrical drill	30.91	1.13	28.61 to 33.20	

p**: Student’s t-test.

For each intervention that required irrigation, the final dentin temperature decreased by a mean of 12.76 °C (95% CI: 14.42 to 11.10), a difference that is statistically significant. Note that irrigation can be considered a thermal modulator during tooth preparation. Regarding the type of bur, the use of a cylindrical bur showed a mean increase in the final temperature (β = 0.59; 95% CI: 0.12 to 1.31), a difference that is not statistically significant. Finally, for every degree that the enamel temperature increased, the final dentin temperature increased by a mean of 0.40 °C (95% CI: 0.21 to 0.58), a difference that was also statistically significant. This relationship shows that the thermal behavior of the enamel has a direct influence on the biogenic heat generated in the dentin, indicating that both dental structures have a certain temperature in their thermal behavior over time. (Table 3)

**Table 3:** Linear Regression Analysis on the Predictors of Final Dentin Temperature.

Variables	Coefficient	Final temperature in dentin	
		β Crude	
		p	IC 95%
Irrigation With irrigation	−12.76	0.000	−14.42 to −11.10
Drill Cylindrical	0.59	0.104	−0.12 to 1.31
Temperature in enamel	0.40	0.000	0.21 to 0.58
Constant	26.11	0.000	20.44 to 31.77

## Discussion

Advances in dentistry have incorporated heat-generating equipment, such as dental lasers, curing lights, and high-speed handpieces^15^. However, the heat produced by these devices can negatively affect the enamel and dentin, as both tissues are vulnerable to the mechanical stress caused by the increased temperature. Furthermore, it can cause inflammation in the tooth pulp, which, if left unchecked, could lead to irreversible damage^15,16^. Thus, water cooling appears to be a necessity in avoiding iatrogenic injuries due to frictional heat. During the course of the cut, the high‑pressure bur should be used without irrigation^17^, the relevance of our study rests on quantifying the magnitude of difference in temperature between enamel and dentin considering irrigated and non-irrigated conditions, and further regression analysis clearly show that enamel temperature accounts to dentin heat accumulation thus giving mechanism to the clinical guideline and supporting irrigation as general means of prevention and a thermal modulator that also preserving pulp vitality.

Our study demonstrated that, in the absence of irrigation, dentin reached significantly higher temperatures (38.7 ± 0.32 °C) than enamel (30.7 ± 2.23 °C), likely due to its lower ability to dissipate heat generated by mechanical friction. This thermal behavior aligns with findings from Lancaster et al., who assessed emissivity using thermal imaging and reported higher values in enamel (0.96–0.97) compared to dentin (0.93–0.94). These results suggest that enamel is more efficient at releasing thermal energy, while dentin tends to retain it—thereby elevating its temperature^18^.

Our findings are also in agreement with a subsequent study by Lancaster et al., who conducted infrared thermographic mapping of dental tissues and found that enamel exhibited greater thermal conductivity than dentin. Under in vitro conditions with healthy tissue, this translated into reduced heat accumulation in enamel and faster heat dissipation compared to dentin^19^. This pattern was mirrored in our results: enamel showed lower temperature increases during preparation, whereas dentin reached markedly higher levels when irrigation was absent. Such consistency across studies reinforces the understanding that dentin’s limited thermal conduction capacity makes it more susceptible to heat retention and, by extension, thermal damage if cooling mechanisms are not used.

Additionally, our results are consistent with those of Soori et al., who measured cavity preparation temperatures in enamel and dentin using infrared thermometry calibrated with thermocouples. Their study also showed higher temperature values in dentin—especially under non-irrigated conditions—which they attributed to dentin’s lower mineral density and higher organic content, factors that impair heat dissipation^15^. Together, these findings highlight the differential thermal behaviors of dental hard tissues and emphasize the importance of effective cooling techniques, such as irrigation, to prevent pulp injury during procedures involving high mechanical friction.

One of the main strengths of our study was the use of premolars extracted for orthodontic indications, which provided greater naturalness and clinical applicability compared with artificial or animal models. Furthermore, conditions with and without irrigation, as well as different types of burs, were included, allowing a systematic evaluation of the generated thermal effect and a direct comparison of the behavior of enamel and dentin. Furthermore, the use of the Fluke thermal imaging camera provided us with precise and detailed measurements of the thermal variations during drilling. However, although the use of extracted human teeth represents an advantage over animal models, working with teeth outside the body limits the replication of real physiological conditions, such as blood flow or nervous system response, factors that can influence heat dissipation. Furthermore, factors in the experimental environment, such as the ambient temperature, may have affected thermographic measurements, compromising the accuracy of the results. Future studies should also attempt to replicate clinical conditions better by assessing pulpal blood flow simulation, standardized irrigation and controlled variables. These would improve consistency and support thermographic data.

## Conclusion

Tooth preparation results in higher thermal effects on dentin compared to enamel both with and without additional water cooling (although the effect was marginally lessened in dentin when additional water cooling was applied), but this was markedly more pronounced under non- irrigated conditions, as evidenced by higher infra-red readings which show dentin is more susceptible to accumulation of frictional heat. Water cooling was justified for thermal safety, consistently lowering temperatures of enamel and dentin whatever bur type was used. Irrigation acted as a true heat modulator, lowering dentin to an average of 12.76 °C below temperature levels found to cause pulpal injury. Regression analysis confirmed that the effect of enamel temperature on dentin temperature reflected the inherent dependence of dentin and enamel heat production on each other. We provide extensive justification for these clinical observations and reinforce the important practical argument that irrigation is critical to pulp protection during preparation.

## Data Availability

All the data presented in this manuscript are available in the Scopus database using the search query in the methodology section. In addition, the data used during the current study are available from the corresponding authors and can be requested from the corresponding author.

## **Abbreviation**

DC: Degrees Celsius
